# Perception of Everyday Sounds: A Developmental Study of a Free Sorting Task

**DOI:** 10.1371/journal.pone.0115557

**Published:** 2015-02-02

**Authors:** Aurore Berland, Pascal Gaillard, Michèle Guidetti, Pascal Barone

**Affiliations:** 1 Unité de Recherche Interdisciplinaire Octogone, EA4156, Laboratoire Cognition, Communication et Développement, Université de Toulouse Jean-Jaurès, Toulouse, France; 2 Centre de Recherche Cerveau et Cognition, Université de Toulouse UPS, CNRS-UMR 5549, Toulouse, France; Kyoto University, JAPAN

## Abstract

**Objectives:**

The analysis of categorization of everyday sounds is a crucial aspect of the perception of our surrounding world. However, it constitutes a poorly explored domain in developmental studies. The aim of our study was to understand the nature and the logic of the construction of auditory cognitive categories for natural sounds during development. We have developed an original approach based on a free sorting task (FST). Indeed, categorization is fundamental for structuring the world and cognitive skills related to, without having any need of the use of language. Our project explored the ability of children to structure their acoustic world, and to investigate how such structuration matures during normal development. We hypothesized that age affects the listening strategy and the category decision, as well as the number and the content of individual categories.

**Design:**

Eighty-two French children (6–9 years), 20 teenagers (12–13 years), and 24 young adults participated in the study. Perception and categorization of everyday sounds was assessed based on a FST composed of 18 different sounds belonging to three a priori categories: non-linguistic human vocalizations, environmental sounds, and musical instruments.

**Results:**

Children listened to the sounds more times than older participants, built significantly more classes than adults, and used a different strategy of classification. We can thus conclude that there is an age effect on how the participants accomplished the task. Analysis of the auditory categorization performed by 6-year-old children showed that this age constitutes a pivotal stage, in agreement with the progressive change from a non-logical reasoning based mainly on perceptive representations to the logical reasoning used by older children. In conclusion, our results suggest that the processing of auditory object categorization develops through different stages, while the intrinsic basis of the classification of sounds is already present in childhood.

## Introduction

One of the most crucial aspects of childhood development is the ability to acquire language and communication [1,2]. Communication is essential during development because children learn to express their needs, emotions, and thoughts. A large body of studies has focused on spoken language and has shown the existence of different stages of speech comprehension during development, ranging from phonemic discrimination to more complex processing, including lexical or syntactic knowledge [3–8]. Numerous studies have explored low-level auditory skills, and these have provided important principles of speech comprehension, such as the perception of intensity, frequency, or temporal differences that compose auditory stimuli [9,10]. Due in part to early ontogenesis, most auditory functions are functional at birth, but some aspects of auditory processing mature relatively early during development, while other features of acoustic processing (e.g., sound localization, hearing in the presence of background noise, and attention) require a longer experience of hearing sounds. Although complex auditory processing, such as music perception development, has also been widely studied [11–14], to our knowledge no study has evaluated how the perception of environmental sounds matures during normal development.

The aim of the present study was to analyze how children with normal hearing of different ages perceive and categorize complex, natural everyday sounds. The principles of “object categorization” have mainly been studied in the visual modality [15]. A categorization consists of defining the properties that are shared by several objects. The combinations of these properties are used to construct classes of objects that are considered similar. In consequence, categorization processes are fundamental in structuring not only perceptual but also cognitive skills. Numerous developmental studies have investigated how categorization processes emerge in the visual domain, and have demonstrated that children acquire several strategies to categorize objects. The most frequent is taxonomic categorization [16], which is a type of grouping based on a shared knowledge of the same common semantic properties, such as the furniture, the animals, the vegetables, etc. In addition to vision, recent studies have been able to show age-dependent taxonomic categorization for olfactory stimuli [17].

Auditory perception remains relatively poorly explored in developmental studies. Research on auditory development [18] suggests that this acquisition evolves according to three main stages linked to the maturation of the auditory cortex and cognitive processing: from birth to 6 months, from 6 months to 5 years, and from 6 years to adolescence. For the perception and organization of sounds in childhood, studies have focused mainly on language [19–24] and, for example, babies are able to categorize different types of vowels of their native language as early as 6 months of age or even earlier [25–27]. The utility of the classification of vowels seems to be evident because of the interest in speech acquisition in children. However, no study has explored how children can categorize natural sounds during their development. In contrast, numerous studies have investigated how adult listeners categorize and perceive auditory environmental sounds (see, e.g., [28,29]). Most of these studies explore how we categorize natural sounds based on pairwise similarity judgments [30–32]. Typically, participants listen to pairs of sounds and rate them on a Likert-type scale with respect to their common properties. When using such an approach, participants tend to group auditory stimuli according to their acoustical parameters [33], but more abstract features, such as emotional content, are unlikely to be taken into account in such similarity judgments. However, semantic-based judgments can be involved in the categorization processes during the task [29]. One alternative method to determine how natural sounds are perceived is to use a free sorting task (FST), a method that offers several advantages, including using a large set of stimuli. FST has been only recently applied in the auditory domain due to the complexity of the implementation [34,35]. In a FST, participants group objects according to their common semantic or acoustic properties. While such a free categorization process is closely related to similarity judgment, the process involves more holistic-based decisions [36] and is more strongly influenced by cognitive factors [37,38]. A recent behavioral comparison study clearly demonstrated the specific advantages (and disadvantages) of applying a FST or dissimilarity rating in the auditory modality [39]. Although the FST is probably the most efficient procedure, as it can be used to test the highest number of stimuli in the fastest time, the FST has lower reliability [39]. This can be partly compensated for by increasing the number of participants. In an auditory FST of environmental sounds, three main features that make up the basis of the different categories can typically be extracted from the studies [40]: the source of the sounds (e.g., animals, vehicles), the context or the location (e.g., outdoor sports), and more abstract-related concepts (e.g., actions, goals, necessities). Also, there is evidence that the human voice or musical sounds, for example, in contrast to environmental sounds, share “the fact that they take advantage of modulations of acoustic parameters specifically for information-bearing purposes” [41], which can be used to build up similarity judgments.

Here we have developed an auditory categorization study for children using a FST. We examined the different classification strategies used at different development stages from 6 years to adulthood to infer the development of the cognitive organization of everyday sounds. In light of the observation of a progressive acquisition of categorization skills in the visual and olfactory domains [16,17], we hypothesize that a similar developmental progression can be observed in the auditory modality. Further, since auditory perception improves during childhood, we hypothesize that the comprehension of complex sounds, as observed by a FST, will also change during development. Lastly, because the FST involves some cognitive aspects (from concept formation to short-term memory), it is highly probable that the sorting strategies performed by the children will evolve during normal development.

## Materials and Methods

### Participants

A total of 82 French children between 6 and 9 years of age participated in the study (Table 1). Children were recruited from two different primary schools and from recreational centers. Each participant had normal hearing and was at an appropriate school level with respect to age. In addition, 20 teenagers (age 12–13 years) and 24 young adults (age 18–30 years) were recruited for comparison. The study was approved by the Comité de Protection des Personnes (CPP) Sud-Ouest et Outre-Mer I (n°11 227 02). Each participant gave their written informed consent, and parents' consent was also obtained for children. All participants reported no auditory, neurological, or psychiatric diseases. All participants had normal or corrected-to-normal vision.

**Table 1:** Description of the participants.

Age Group (years)	*n*	Average age (years± sd)	Min Age (y;m)	Max Age (y;m)	Gender (Female / Male)
6	22	6.5 ± 0.25	5;11	6;11	10 / 12
7	22	7.6 ± 0.22	7;1	7;11	9 / 13
8	22	8.4 ± 0.24	8;0	8;9	12 / 10
9	16	9.3 ± 0.26	9;0	9;11	9 / 7
12–13	20	12.6 ± 0.52	12;1	13;10	13 / 7
18–30	24	23.6 ± 0.34	18	30	13 / 11
Total	126				66 / 60

### Materials

The task consisted of an auditory FST presented via the open-source software TCL-LabX [42]. We defined three a priori categories of sounds similar to those used in previous studies: non-linguistic human vocalizations, environmental sounds, and musical instruments [30,32,43–46]. Eighteen sounds (Table 2) were selected from a larger set of 45 sounds (http://www.freesound.org/) through a pilot test performed on a separate group of 15 French adults to assess the recognition, familiarity, and typicality of each sound [47]. Pre-testing ensured that the selected sounds were easily identified and were prototypical for their category. All stimuli were monophonic and were recorded in .wav format with a sampling frequency of 44,100 Hz. They were normalized in duration (2 seconds) and loudness before being imported into TCL-LabX.

**Table 2:** Stimuli included in the FST grouped in ***a priori*** classes.

Human non-linguistic vocalizations	Child	Babbling
		Tears
	Woman	Cough
		Laugh
	Man	Yawn
		Scraping throat & cough
Environmental sounds	Alert	Horn
		Bell
	Animals	Bird
		Cow
	Everyday sounds	Front door
		Rustling of paper
Musical instruments	Stringed instruments	Violin
		Double bass
	Wind instruments	Traverse flute
		Tuba
	Percussions	Kettledrum
		Drum kit

The selected sounds were complex and were probably difficult to classify based only on physical criteria. Due to the large variability in the sounds, the frequency values, frequency bandwidth, and basic acoustical features (pitch, duration, intensity) were insufficient to distinguish each sound [48]. As the hypothesis is based on the comprehension of complex sounds, we chose our selection of sounds using a semantic approach and not an acoustic one. However, taking into account that acoustic characteristics give important cues to identify and understand the sounds, we analyzed each sound individually. We extracted four acoustic parameters (fundamental frequency, spectral center of gravity, width of the spectrum, and the presence of harmonic sounds in each sound sequence) and three subjective parameters (the number of different sounds, the resonance, the sound length). Most of the acoustic characteristics were obtained using PRAAT software. The spectral center of gravity is computed using the PRAAT algorithm. The spectral center of gravity is the average frequency of a sound weighted by the power of the spectrum (amplitude), and is often used to measure the "brightness" of a sound. The presence or absence of the harmonicity in the sounds is assessed in the same way using PRAAT and by performing a short-term HNR analysis. The resonance is a perceptive indicator of the presence or not of the resonance of the sound (as a perceptible decay of the sound as a function of time). Although some of these parameters were related to the a priori classes made before testing the participants—for example, the presence of harmonics for non-linguistic human vocalizations—most of them were not relevant to effectively define the categories made by the participants, such as the association “bell door and door” (see below). To confirm this hypothesis, we applied statistical tests to the acoustic values obtained in each a priori class. First, an analysis of variance (ANOVA) performed with the quantitative acoustical parameters of the sounds as the dependent variable and the three a priori classes as the between-subjects factor, revealed no differences between a priori classes concerning the fundamental frequency [F(2,14) = 2.05, p = 0.17, η2 = 0.23], or the width of the spectrum [F(2,15) = 0.34, p = 0.72, η2 = 0.04]. The presence of harmonicity was also found to be similar for the three classes (χ^2^ (2, N = 18) = 2.57, p = 0.276). Moreover, while an analysis of variance performed on the spectral center of gravity revealed a marginal statistical difference [F(2,15) = 4.02, p = 0.04, η2 = 0.35], post hoc tests showed that the significance was limited only to the differences between the musical sounds and the environmental sounds (Scheffé, p < 0.05). These preliminary analyses tend to suggest that the basic acoustical parameters of the sounds cannot fully explain the definition of our three a priori classes.

**Fig 1:**
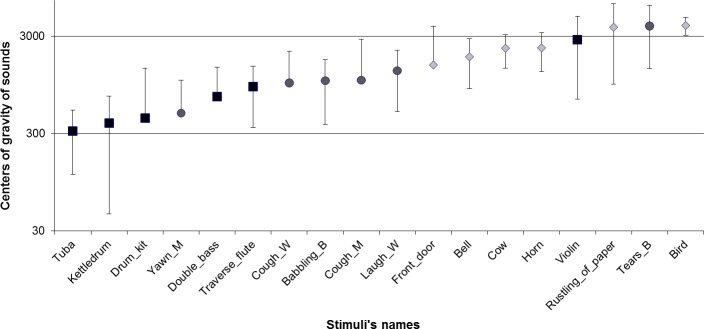
Distribution of the center of gravity of the sounds and frequency bands, ranging by frequency from low to high (scaled in log to base 10). Circles represent non-linguistic human vocalizations, square musical instruments, and diamond environmental sounds.

Fig. 1 illustrates, for instance, the spectral center of gravity and frequency band of the 18 sounds with respect to the three a priori classes. In this example, each class is composed of sounds of various spectral centers of gravity or frequency bands. Sounds of similar acoustic features are observed in different classes. Thus, it is unlikely that a categorization during the FST will lead to a partition similar to the a priori classes based only on the acoustic characteristics.

### Procedure

Participants sat in a quiet room facing the computer. The 18 sounds were aligned on the computer screen as represented by 18 numbered and colored buttons. The positions of the buttons on the computer screen were placed randomly, but were fixed for each participant. The sounds were delivered through a headphone plugged into the computer.

Participants received instructions to group the sounds according to their own criteria: “Put together the sounds that go well together. You can do families of one, two, three, or more sounds, as you wish.” No further instructions were given concerning the categorization criteria, and participants were allowed to create as many groups as they wished. Thus, a category may contain only one stimulus or even all the stimuli. The participants listened to the sounds as many times as they wished by clicking on the individual buttons. Grouping of sounds was performed by moving the associated buttons using the computer mouse. Participants were allowed to change the groups of sounds they made as many times as they wished. No time constraint was applied, and the duration of the experiment was not limited. Once participants decided that the categorization was complete, they were not allowed to modify it.

At the end of the test, the participants were asked to define and name the categories they made. They were allowed to listen again to the sounds in order to name each category.

In all cases, the experimenter was seated near the participants to help them manipulate the computer mouse if needed. In fact, in most cases, even the youngest children were able to move the squares on the screen and group the sounds together easily. Therefore, we believe that the technical aspect of the task or the level of computer skills of the children did not influence the strategy of sounds categorization.

### Analyses

Two levels of analyses were performed. First, we examined the participant’s actions performed during the categorization. Data included the number of categories and the number of times the participants listened to the sounds. The normality of the distributions was then evaluated with a skewness and kurtosis test using SPSS 20.0.0. To analyze the global effect of age on the actions performed by the participants, ANOVA was applied and a post hoc Scheffé test was used to compare values across the different age groups.

Next, we analyzed the categorization strategies adopted by the participants. We evaluated the structure and the content of the categorizations, applying a central partition analysis to identify a possible consensus opinion on the classification. The consensus partition represents the average of all the possible partitions. Indeed, a partition is defined as consensual when a minimum of 50% of the subjects grouped at least two sounds together [49]. This average partition shared by the participants is obtained by computing the number of co-occurrences of sounds within groups. The result is a matrix with positive and negative values. Positive scores correspond to the pairs produced by more than 50% of the participants. These positive pair scores allowed us to calculate the weight or the optimal score of the partition corresponding to the sum of all the positive pair scores. The homogeneity of the opinions of the groups is determined by the optimal score of the partition multiplied by the total number of opinions (i.e. the general score obtained by computing the total number of cells in the co-occurrence matrix by the number of subjects). The higher the general score is, the more the consensus partition represented the homogeneity of the opinions of the groups [50]. If there are too many singletons (i.e., classes composed of only one sound) in the obtained partition, no common opinion can be extracted for this specific object. The homogeneity score is consequently weak. So, to obtain the more homogeneous common opinion(s) performed by the entire set of participants, all the possible between-subjects associations have to be analyzed. It is then possible to obtain several consensus partitions, dividing the population into different groups of opinions.

Moreover, a dissimilarity analysis was computed with the distances between the different stimuli. An aggregated dissimilarity matrix Δ = δ(ij) indicates the distances between each sound [51]. It represents the sum of the individual dissimilarity matrices. In those matrices, the lower the distances are for each participant and each sound pair (i, j), the more the sounds have been put together. A distance value is close to zero if the sounds were grouped in the same category, and a distance value close to 1 indicates that the sounds are segregated from each other. As a result, the aggregate dissimilarity between the sounds i and j (i, j = 1,2,…P) was evaluated by the number of participants who did not put two sounds into the same group.

Further, we constructed additive trees to graphically represent the matrix of the distances resulting from partitions emanating from the age groups [52–56] using the AddTree algorithm [56]. This type of representation has been applied to semantic categorization tasks [57,58], an acoustic categorization task [35], and to represent hierarchical organizations of knowledge [59]. Distance trees provide a representation of the dissimilarity between two sounds expressed by the length of the edge (topologic criteria). The shorter the line connecting the two sounds was, the more the sounds were judged to be similar and vice versa. The tree-like graphic is completed by topologic and metrics criteria that is used to assess the robustness of the graphic representation [60]. The topologic criteria are expressed by the percentage of well-represented quadruplets and by the arboricity, which corresponds to the correlation of the distance between two objects (calculated from the partition of each participant) and its representation on the additive tree. Only correlation values greater than 0.75 can be considered as reflecting a strong reliability of clustering between two sets of stimuli [55]. The metric criterion “stress values” is used to compare the calculated distance and its tree representation, and provides quantification of the graphic representation that minimizes the within-group variability. Indeed, it allows us to evaluate the quality of the representation of the tree, particularly the relevance of the presence of a node and its supposed cognitive representation (a category). The stress index represents the reliability of the graphic representation and values above 0.15 can be considered as robust (i.e., above 85% of variance explained). Thus, the distance-tree represents the links (the fact that two sounds have been put together by a majority of participants) and the structure of this categorization (sound categories made by participants).

We hypothesized that the participant's age affects the listening strategy and the category decision, as well as the number and the content of individual categories.

## Results

### Participants' actions: Listening strategy

We analyzed the listening strategy of the subjects by comparing how many times the subjects in each age group listened to individual sounds. Further, we determined whether some sounds or categories of sounds were listened to more frequently than others. A mixed-design analysis of variance with age groups as the between-group factor (six levels: 6; 7; 8; 9; 12–13 years old; Adults) and the type of sounds as the within-participants factor (three levels: non-linguistic human vocalizations; musical sounds; environmental sounds) showed a significant effect of age on the average number of times that sounds were listened to [F(5,120) = 9.327, p < 0.001, η2 = 0.28].

**Fig 2:**
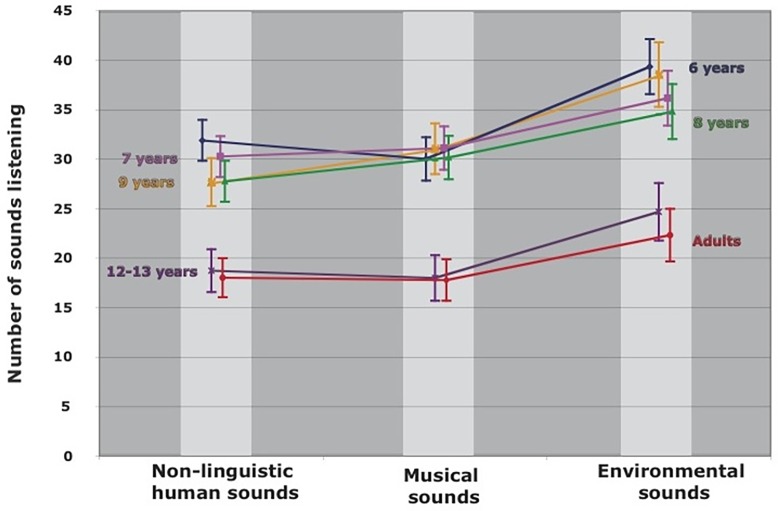
Average number of sounds listening as a function of age and of the *a priori* category. There are a significant differences between age groups and categories.

As shown in Fig. 2, the average number of listens was about two times higher in the younger children (6–9 years) compared to the teenagers (12–13 years) and adults (18–30 years). The post hoc tests confirmed that the listening strategy of older children and adults was significantly different to that of younger children (Scheffé, p < 0.05 in all cases), while listening scores were similar between teenagers and adults (Scheffé, p = 1). The mixed-designed ANOVA also revealed a significant effect concerning the listening frequency of sounds according to their a priori group [F(2,240) = 49.512, p < 0.001, η2 = 0.29]. As clearly illustrated in Fig. 2, the subjects listened many more times to the sounds that belong to the environmental category. Such a tendency can be observed in each age group, as the mixed-designed ANOVA did not reveal an age-category interaction [F(10,240) = 1.124, p = 0.344]. For example, the sound of paper rustling was the most listened to by the participants (more than six times across age groups), probably because it was more difficult to recognize. In contrast, the sound of a baby crying was the least frequently listened to by the subjects (about three times on average) as the sound was clearly identifiable. Moreover, human sounds and musical sounds presented a similar frequency of listening in all age groups.

### Participants' actions: Number of classes

**Fig 3:**
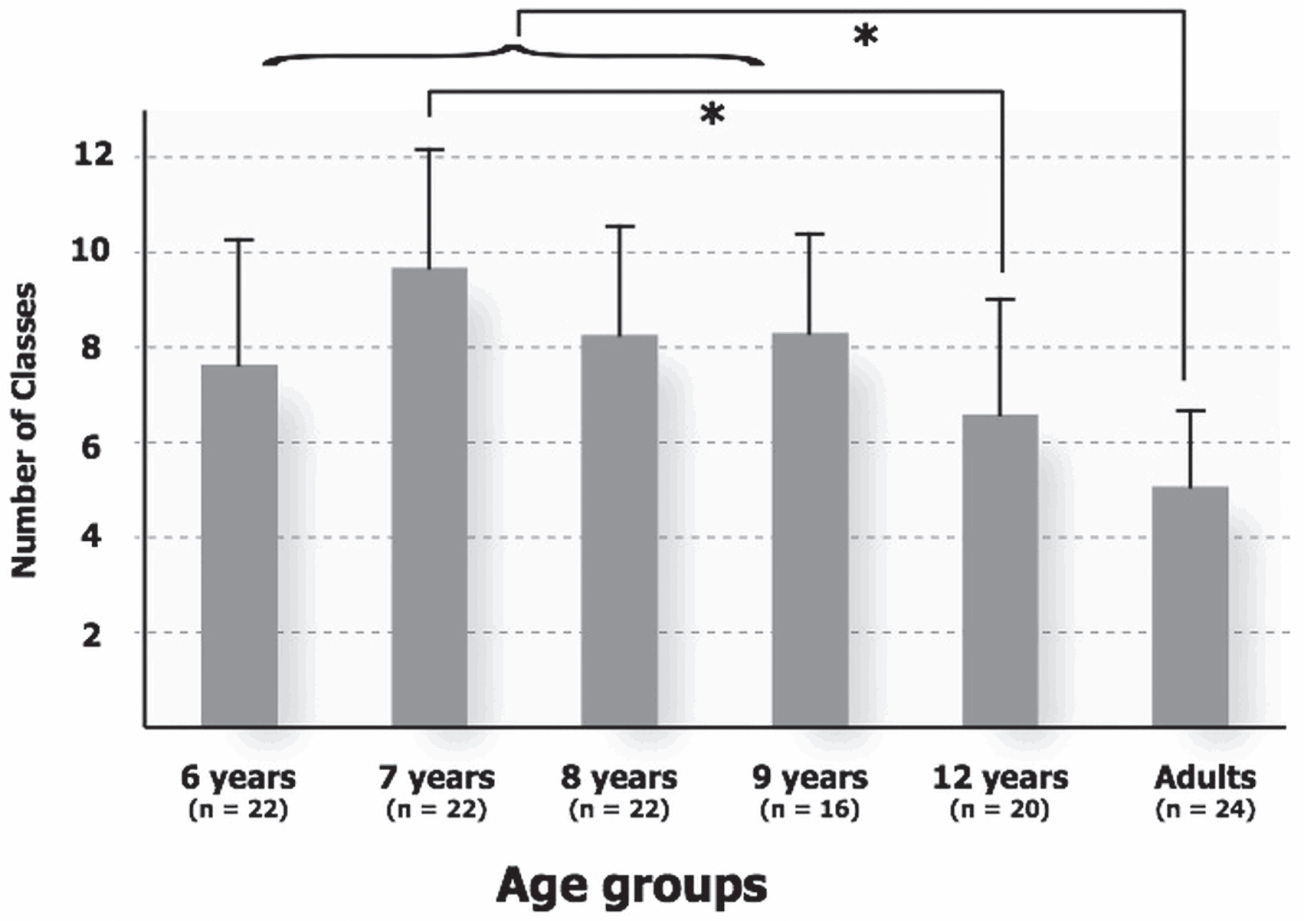
Average number of classes as a function of age of the participants. Standard deviations are depicted for each age group. Significant differences between age groups are represented by the brackets and astericks (representing p<.05).

The number of classes performed individually by each subject ranged from a minimum of two to a maximum of 17. Six tables, added as (cf. S1 and S2 Tables), show how individual subjects grouped together each sound object: in these tables, each row corresponds to a sound, each column corresponds to a participant, and the numbers in each cell correspond to the group in which the sounds have been put by the corresponding participant. As illustrated in Fig. 3, the average number of classes varied as a function of age of the participants, and the greatest number occurred for the 7-year-old group (9.7 ± 2.5, mean ± SD) and the fewest for the adults (5.1 ± 1.6, mean ± SD).

We ran an analysis of variance with the number of classes as the dependent variable and age groups as the between-subjects factor (six levels: 6; 7; 8; 9; 12–13 years old; Adults). It demonstrated a significant age effect [F(5,120) = 11.027, p < 0.001, η2 = 0.32]. Post hoc tests showed a significant difference between the numbers of classes produced by the children (6–9 years old) and by the adults (Scheffé, for the 6-year-old group, p < 0.05; for all other comparisons, p < 0.005), and thus children built significantly more classes than adults. Teenagers (12–13 years old) showed an intermediate strategy (6.6 ± 2.42, mean ± SD) that was not significantly different from that observed in younger children (p = 0.823 with the 6-year-old group) or adults (p = 0.439) and represented a transitional stage for this categorization feature. Further, we observed a decrease in the variability in the strategy with increasing age as expressed as a decrease in the standard deviation (from 2.63 at 6 years to 1.586 for adults). A Levene's test (or F-test), performed to assess the equality of variances, that is, to evaluate whether standard deviations of the different groups were significantly different or not, revealed a lower standard deviation in adults, thus reflecting a higher consensus in the strategies used, compared both to children between 6 and 9 years of age (F = 4.66, p = 0.033) and to teenagers (F = 8.08, p = 0.007).

### Categorization type

**Fig 4:**
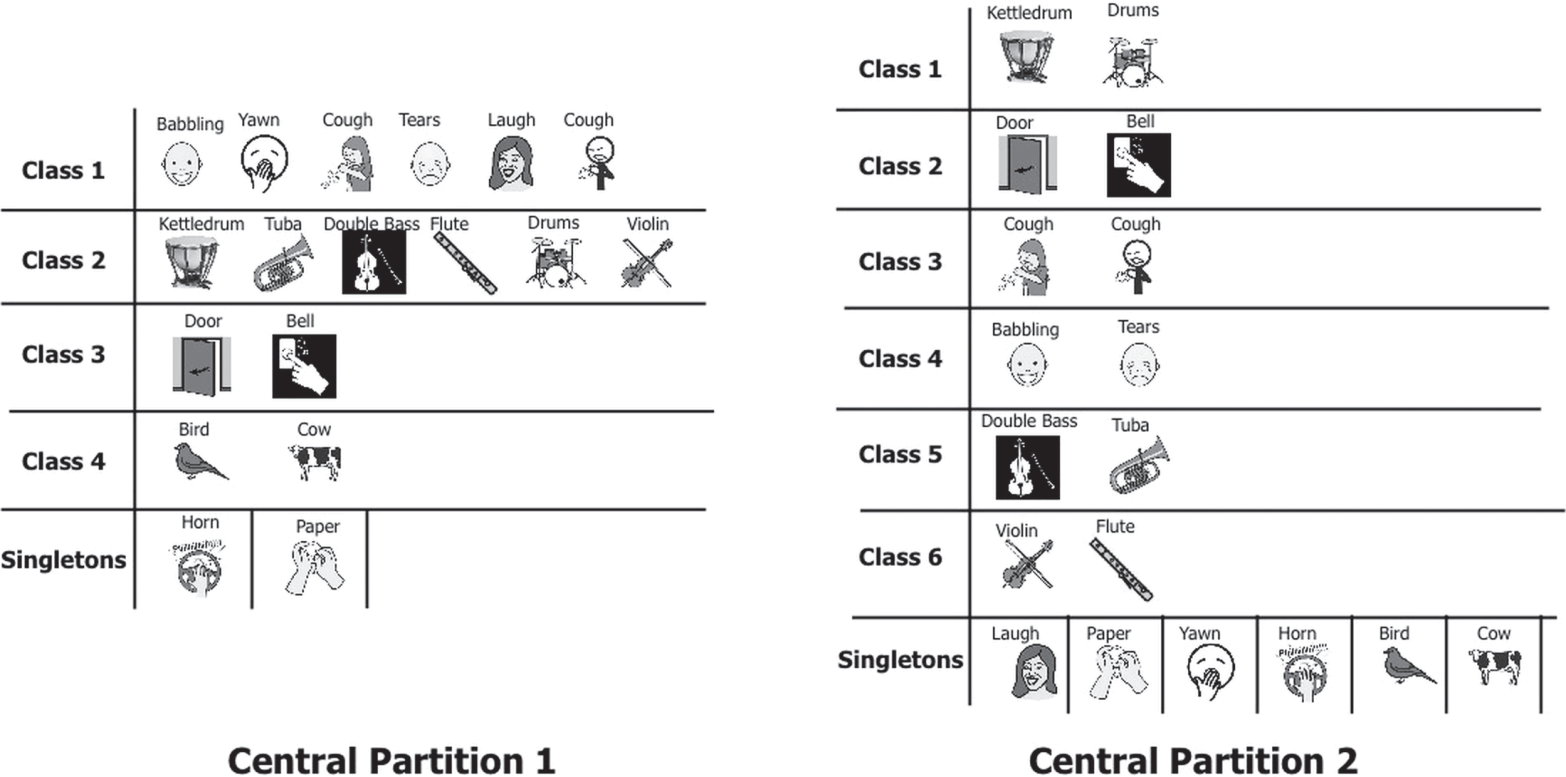
Illustration of the two main central partitions performed by participants, independent of the age.

**Fig 5:**
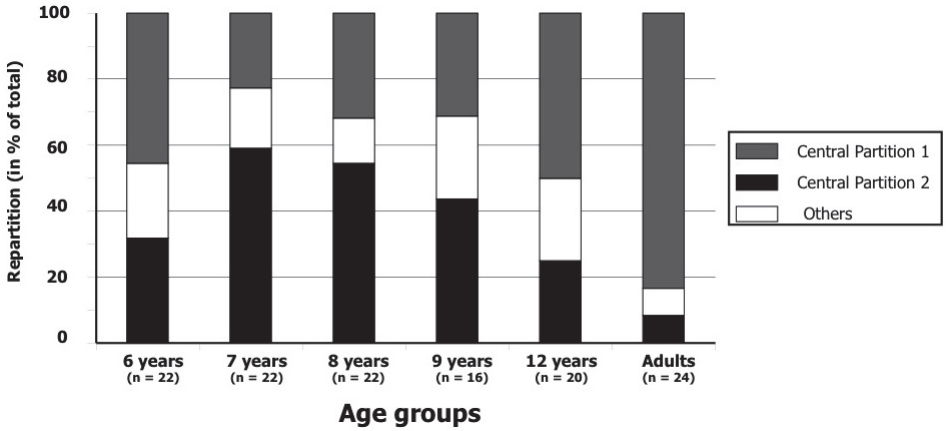
Proportions of Central Partitions 1 and 2 performed by the subjects according to their age. The other types of partitions have been grouped together (labeled “Others Partitions”).

**Central partitions.** We first considered all 126 participants without any distinction of age, but for this set of subjects we did not find a single partition consensus. As explained in paragraph 2.4 above (p. 10), there were too many singletons in the obtained partition, so the homogeneity was weak and no common opinion could be extracted. We then tried all the possible between-subjects associations. Two partitions emerged from the 126 classifications as being the more homogeneous common opinions that were performed by the entire set of participants. These two partitions represent partitions in which the sum of the distances to the individual partitions is minimal (see [50] for more details). These two main categorizations of the auditory stimuli differed both in the number of classes that composed them (Fig. 4), and in terms of the age group that principally followed this grouping (Fig. 5).

Central partition 1 contained two singletons (i.e., two classes composed of only one sound) and four classes of sounds (two classes containing six sounds each, and two classes containing two sounds). At a semantic level, the two main classifications of this central partition were related to the human sounds and to the musical sounds. Environmental sounds were distributed within the remaining classes.

Central partition 2 was mainly composed of pairs of sounds, as there were six classes of duets in addition to six singletons. At a semantic level, each pair of sounds belonged to the same taxonomic classification: human sounds, musical instruments, and environmental sounds were not mixed.

Central partition 1 was generated by nearly half of the participants (57 out of 126 participants). Participants in all age groups produced this partitioning, but 83% of adults performed this type of categorization (Fig. 5). On the other hand, only 23% of the 7-year-old group produced this central partition.

Central partition 2 was generated by 46 participants (36%). This partitioning was produced predominantly by the 7- and 8-year-old groups. Only a few adults (8%) produced this central partition 2.

These results highlight some differences in strategies according to age. χ^2^ tests performed on the repartition of the subjects in the partitions in each age group showed that the distribution of the subjects in each central partition is significantly different between children and adults (all comparisons, p < 0.05). The first central partition was produced significantly mostly by the adults and the second mostly by the children. Fig. 5 reveals a progression in the evolution of the categorization approaches: children 7–9 years mainly used comparisons in pairs, while teenagers between 12 and 13 years and adults appear to present a more generalized approach by producing bigger classes. The youngest group (6 years old) produced the central partitions 1 and 2 similarly (45% and 32%, respectively); they produced heterogeneous classifications with a bimodal distribution.

**Fig 6:**
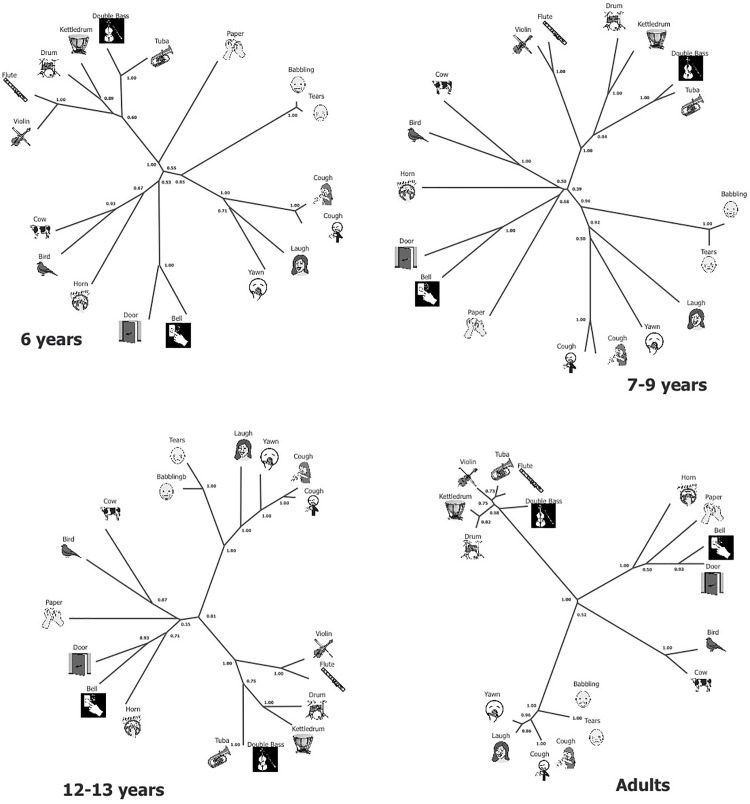
Additive trees showing both similarity and differences across sounds as function of age. The shorter is the line connecting two sounds, the more the sounds are judged similar.

**Classification mechanism and class content.** We computed additive trees of the sounds to graphically represent the categorization content of the participants in each age group, based on the aggregated dissimilarity matrices. These additive trees allowed us to objectively quantify the relationships that link each auditory stimulus in a given partition. In agreement with the results based on the central partition analysis, the additive trees appear to differ significantly when comparing each age group (6, 7, 8, 9 years old, teenagers, and adults; Fig. 6). Metric criteria allow us to base our understanding of the structure of the categorization on the tree representation reading, as indicated by a large proportion of explained variance (95%, 96%, 94%, 93%, 92%, and 95%, respectively). We note that the variance is constant across ages. Metric criteria were confirmed by topological criteria. Indeed, we observed 89%, 84%, 89%, 83%, 94%, and 93%, respectively, of well-represented quadruplets, and arboricity values ranged from 0.76 to 0.91 (0.82, 0.83, 0.79, 0.76, 0.85, and 0.91, respectively, for 6, 7, 8, 9 years old, teenagers, and adults). These values ensure a robust representation of the data [60–62].

Concerning the additive tree observed in the 6-year-old group, it presents an intermediary product between that observed in the 7–9-year-old children and that observed in the adults. The tree revealed four main categories and the distances inside each category are long (mean 20.6), except for the baby voices (mean 1.1), the two human coughing sounds (mean 2.3), and the two melodies (violin and flute, mean distance 4.7). This result highlights the high inter-individual variability in the class contents as previously discussed. However, in spite of a heterogeneous categorization, the additive tree presents robust metric (stress index = 95%) and topological criteria (arboricity = 0.82). The first category grouped six items (arboricity = 0.85), which corresponded to non-linguistic human vocalizations. This category can be subdivided into two well-differentiated subcategories: adult sounds (arboricity = 1) and baby sounds, which are very close to each other. The second category consisted of a grouping of musical instruments that included the sound of rustling paper (arboricity = 1). Again, this large group is subdivided into three categories and one singleton: melodies (violin and traverse flute; arboricity = 1, mean distance = 4.71), held notes (tuba and double bass; arboricity = 1, mean distance = 5.26), percussions (kettle drums and drum kit; arboricity = 0.89, mean distance = 7.48), and rustling of paper. The third category grouped animal sounds (arboricity = 0.93; mean distance = 13.94), and the fourth category contained the front-door and bell sounds (arboricity = 1; mean distance = 9.24).

Between 7 and 9 years old, we found five classes, which were all subdivided into subcategories. Associations are essentially realized two-by-two, as shown with the central partition analysis for this age range, and means distances inside each significant pair decrease between 7 and 9 years old, which indicates that homogeneity of the categorizations increases with age.

The majority of teenagers produced three blocks of sounds: the human voice, musical sounds, and animal sounds. As in the adult categorization frame, baby noises were more often associated with other human non-linguistic vocalizations, but this was not observed in the younger age groups. Other sounds are still not “well” classified and form isolated singletons. The differences between teenagers and adults concerned the environmental sounds. The adults grouped the horn and the rustling of paper with the bell and the door opening, while the horn and the sound of rustling paper tended to form isolated singletons in the 12–13 years group. Further, the additive tree of the teenagers group can be clearly distinguished from the one observed in younger children (7–9 years) because teenagers abandon the classification by pairs to present a higher generalization approach by grouping more sounds together (two classes of six sounds).

In the adults, we observed topography of the tree in which sounds were grouped in classes that were close to the a priori ones. In the adults’ trees, we observed four well-separated edges, which indicate the presence of four well-defined classes of sounds in a very homogeneous pattern. The homogeneity is expressed by the values of the tree distances between each grouped sound, which are very short or null (mean of distances in all categories = 7.86).

**Quantitative analysis of distances.** In order to quantify precisely the differences in strategies performed by the subjects, two analyses were applied to the categorization results. First, we computed the distances that separated each sound object inside the categorization trees performed by a group of subjects (see Table 3). As explained l. 219, the lower the distances for each participant and each sound pair are, the more the sounds have been put together.

Considering the overall average distances, ANOVA, with the distances between objects as the dependent variable and age groups as the between-subjects factor, showed a significant decrease during development [F(5,264) = 11.102, p < 0.001, η2 = 0.17]) from high values in 6-year-old children (mean 16.32 ± 4.87) to lower distances in adults (mean 10.17 ± 9.08). The decrease in distance is particularly evident from the age of 9 years (Scheffé post hoc test, p < 0.05 for all comparisons). When such distance values are compared with respect to the a priori classes (music, human voice, and environmental sounds), we also observed a significant decrease for both music and human voice stimuli. Post hoc tests demonstrated that teenagers and adults grouped the non-linguistic human vocalizations as in our a priori classes more frequently than the younger children (Tukey, p < 0.05 for all comparisons). Musical sounds were grouped as in our a priori class in a more homogeneous way by the adults than all the other age groups (Tukey, p < 0.05 for all comparisons). However, the distances that separate the objects composing the environmental sounds a priori classes remain the same across ages. Together, these data confirm that the categorization performed by the children evolves during development, being progressively more homogeneous and increasingly similar to that observed in the adults group.

**Table 3:** Average sorting sounds distances.

	Average sorting distance within each of the three *a priori* classes						Average sorting distance between all sounds	
	Non-Ling. Human Voc.		Musical Sounds		Env. Sounds			
	*m*	*SD*	*M*	*SD*	*m*	*SD*	*m*	*SD*
6 y	16.78	7.29	11.24	3.31	20.95	4.41	16.32	4.87
7 y	19.10	6.67	14.98	4.80	21.09	3.69	18.39	3.11
8 y	16.77	6.08	13.77	4.82	20.60	3.52	17.04	3.42
9 y	13.42	5.59	9.64	3.44	15.87	2.76	12.97	3.14
12–13 y	10.82	4.35	10.26	3.93	17.18	2.72	12.75	3.85
Adults	4.91	2.46	4.95	1.65	20.66	8.91	10.17	9.08

Secondly, we assessed whether the categorization strategies were performed in a homogeneous way inside each age group and/or differently during development. For each age group, we computed the distances between subjects obtained from the aggregated dissimilarity matrices of subjects. The aim of this analysis was to seek for “abnormal” behavior or “a-specific” categorization strategies. The presence of “outliers” was defined as subjects presenting, in their age group, inter-subject distances equal to or above 2 SD from the mean distance. In our population, we found only three “outlying” subjects coming from three different age groups: one in the 6-year-old group, one in the 8-year-old group, and one in the 12–13-year-old group. However, to test the robustness of our previous analyses, we compared the differences between the object-distances in each age group, with and without the presence of outliers with t-tests. We found that exclusion of these three atypical subjects did not significantly change the distances distribution in the corresponding age group (all three tests, p > 0.05). From this analysis, we can state that the categorization trees obtained from each age group are quite homogeneous and can be considered as representative of the corresponding age.

## Discussion

The purpose of our work was to understand the nature and the logic of the construction of auditory cognitive categories for everyday sounds during late development. We hypothesized that we would find an age effect on the listening frequency of sounds, on the number of classes produced, on the classification strategy used, and on the type of classes realized.

### Listening frequency of sounds

Our results provide evidence for an age-related effect on the average number of times participants listened to the sounds during the FST. The need to listen several times to the sounds decreased significantly with the age of the participants between childhood (6–9 years) and adolescence (12–13 years) or adults. These differences can be explained by the cognitive process involved in the FST. Auditory categorization involves not only perceptual processes and cognitive representation, but also requires access to the working memory in order to compare the 18 sounds. Such access to short-term memory could explain why children need to listen to the sounds more times due to some lower efficiency [63]. In agreement with the “cognitive economy” principle [57,64,65], the task would become easier for adolescents due to the lower cognitive processing cost [66]. However, the frequency of listening to sounds is also linked to the sound-identification skills. It is manifest that our knowledge of our sensory world progresses during development and, in the auditory FST, sound categorization may rely on sound identification [67]. Again, such a skill is directly linked to the memory abilities, as it has been shown that sound identification is dependent on long-term auditory memory [68].

### Average number of classes and classification strategies

Our results show that the number of classes produced by the subjects is strongly dependent on the developmental stage. Indeed, children produced significantly more classes than adults. This result is directly linked to the categorization strategies.

The 6-year-old children represent a heterogeneous group regarding their construction of classes. About 45% of these children made large classes containing numerous sounds, whereas 32% adopted a sorting strategy by pairs. In both cases, they were not able to systematically provide a clear explanation for the reasons for such groupings. In contrast, older children presented more homogeneous strategies, and the free sorting was mainly built upon classes of no more than two items. While we have not tested children younger than 6 years old, such heterogeneity in the strategies of the FST suggests that this age constitutes a pivotal stage linked to brain maturity [69–73]. This is in agreement with previous studies [63] postulating that there is a radical change between the non-logic reasoning of the 6/7-year-old children (pre-operative stage) and the logical reasoning of older children (operative stage). However, the limit between pre- and post-operative stages is not sharp [69], and pre-operative children might process via logical structures, and depending on the task, some lack of inhibition could be misinterpreted as a failure in logical reasoning [69]. Others have proposed that the transition at 5–7 years old corresponds to a coordination of the fragmentary knowledge that was previously acquired [74], a process that would probably affect cognitive processes such as the FST.

Furthermore, the differences between 6–7-year-old children and older children could correspond to a schooling effect. Indeed, because learning is taught through language, social convention, and education [75], schooling can affect cognitive strategies [76,77]. In the French system, the 6–7-year-old children enter school where they acquire specific instructions according to the scholar norm. At this age, children learn to develop analytical capacities using analogical comparisons, particularly during language acquisition [78]. Indeed, in France, reading acquisition, which begins at about 7 years old, is mainly based on the association between a phoneme and its written form. Such a system, based on a two-by-two association, could explain why children at this age make more classes of pairs of objects. Moreover, many learning processes are based on matching tasks (e.g., memory games). Thus, we suggest that at the age of 6–7 years, children tend to abandon the intuitive classification strategy based on perceptual processing [63] to adopt a matching task strategy (for review, see [79]). Lastly, the differences in the number of classes performed by the subjects, such as in the 7–9-year-old group, could be due to the cognitive cost of the task. As mentioned previously, short-term memory processes are engaged during the FST. As the participants progressed in the task, they used internal representations of the classes that they had previously built to remember the sounds that constitute them. The binary association, which may be induced by the initial stimuli choice, allows a one-to-one comparison between two elements, which is easier than a multiple comparison of several elements [63,80].

Most of the teenagers tended to create larger classes of four or more items. Based on the experience of processing environmental sounds in real situations (implicit learning), they acquired strong skills in sound discrimination to identify the basic level of categorization as well as the subordinate or the superordinate level [65]. The perceptual strategy developed at younger ages (7–8 years old; [63,81]) is preserved throughout the development in conjunction with the logical (or taxonomic) class processing [82–88]. As the goal-oriented perceptual interactions with the environment vary as a function of developmental age, teenagers can build their categorization according to the use necessity. This strategy corresponds to the increase in abstraction capabilities as previously suggested [63]. Lastly, adults produced the smallest number of classes compared to children and adolescents. The adult FST strategy probably relies on internal models (i.e., prototypes; [65]) that are semantically broader and so even more economical in performing the task. Thus, a prototype would not be a consequence of the perception, but an implicit tool that could become explicit for the adolescents and the adults in the choice of the “right prototype” according to the task.

### Types of classes produced

We noticed that, although the initial instructions were identical for each age group, children at each age used association criteria that were slightly different from those used by adults. This could clearly be found in the verbal explanations they gave regarding the categories they made. Firstly, it is important to note that about 10% of the 6-year-old children were not able to provide any justification of their categorization, while this was nearly absent in adults. Further, in the 6-year-old group, children tended to tell stories based on their own experience or vivid events to verbally explain their clustering in about 20% of cases, while again, such behavior was almost absent in the adults (less than 2%). For example, one child associated the horn and the cow because “they horn so the cows on the road do ‘moo’!”. These children mainly used scripts, a free sorting strategy described as figural or schematic [81]. Children aged 7–9 years followed a semantic strategy according to an adjacency principle: percussions, held notes, melodies, baby sounds, and coughs. As in object-matching tasks [89,90], their sorting was based on perceptual and abstractive similarities, which facilitates the access to taxonomic relationships. Adolescents, similar to adults, categorize the sounds into larger classes: the voice, music, and animals, and began to describe them with a more defined label, for example, “musical instrument” or “human sounds.” They are able to classify all the sounds with more abstract and verbally accessible criteria. Indeed, the more the language becomes precise, the more the mental representations become complex [91,92]. Language development facilitates the abstraction of common properties of distinct objects, leading to a de-contextualization of categories [81]. However, figural categorizations do not disappear after 7–8 years old [63,81], and, in some cases, scripts or event schemas are observed until adolescence as, for example, the association of the bell and door sounds. In conclusion, our results suggest that the processing of auditory object categorization is not linear during development, and several strategies of categorization can co-exist within the same developmental stage, as previously reported in other modalities, especially during visual categorization ([82–88]; for a review, see [93]).

While we observed some changes in the free sorting strategies between each age group, our results highlight the semantic proximities in the content of the produced categories, as previously reported [35]. On the one hand, some sounds were never grouped together in any age group, for example, the animal sounds and the musical instruments. On the other hand, to some extent, some stimuli were systematically grouped together. This was specifically the case for the non-linguistic human vocalizations, which were grouped together from the very youngest age. This result is particularly relevant in light of the specific status of the human voice, especially during early developmental stages [94]. The integration of the information from the voice is important for our social communication. The human voice carries not only speech information, but also non-speech identity information, such as gender, age, physical factors, and emotions. Because of the variety of such information derived from the voice processing, the concept of the "auditory face" is now broadly accepted [95]. Further, brain imaging studies have confirmed that there are circumscribed brain regions in the temporal lobe that are specifically involved in processing voice information [96,97]. Of interest, such sensitivity to the voice emerges very early during development, between the fourth and the seventh month after birth [98]. Children can therefore be considered as experts in voice processing (speech and non-verbal vocalizations). However, during the FST of the youngest children (up to adolescence), the baby-voice stimuli were clearly dissociated from the other adult-voice sounds. Young children could identify themselves with the baby voices, whereas the adult voices were considered as another human class. According to the lexical hierarchy [99], the superordinate level used by human adults appears not to be operant in the youngest children.

Musical instruments were also grouped together even in the youngest age group, which made classes similar to that produced by most of the adolescents. However, they conceptualized the sounds differently compared to the adolescents and adults. The 6-year-old children could not identify every sound with the appropriate lexicon (violin, etc.), but they refer to the use of the sound using the general denomination of “instruments” or “musical instruments.” The superordinate abstraction level [64,65] is already present from the perceptual dimension, whereas the basic lexicon has not already been built. This observation appears to contradict the conclusions of previous studies of Rosch based only on the use of the lexicon. This would tend to suggest that, in the perceptual field, the acquisition of the different levels of abstraction seems to be processed differently compared to the acquisition of the lexical abstraction levels.

## Conclusion

The free categorization task of environmental sounds is still a poorly investigated domain [35,38,45,100,101]. However, the analysis of everyday-sound categorization is a crucial aspect of the perception of the surrounding world. Moreover, to our knowledge, no study has investigated this perceptual domain during children's development. The free categorization of sounds allows us to highlight an age effect on how the participants accomplished the task (listening frequency of each sound, number of classes, strategies of classifications used). However, a close examination of the type of class revealed that the children and the adults tended to adopt a similar listening mode [102–104] that allows them to define semantic similarities between everyday sounds. While different, it suggests that the intrinsic basis of the classification of sounds is already present in childhood. While the FST is not commonly used in cognitive representation studies, it represents a particularly interesting approach to understand what makes sense for each participant in the proposed complexity. Our results could constitute a heuristic background to study different populations of patients suffering sensory or cognitive impairment.

## Supporting Information

S1 Table Individual data of FST. Results for 6, 7 and 8 years old participants. Each column represents the classification performed by an individual subject, and in each line, we are providing the category number in which the individual sounds have been pooled together. In consequence, for each individual, the highest number corresponds to the maximum number of classes performed by the subject.
(TIFF)

S2 Table Individual data of FST. Results for 9, 12–13 years old participants and adults. Same convention as in S1 Table.
(TIFF)
