# Supplementary figures and images for: Perception of Everyday Sounds: A Developmental Study of a Free Sorting Task
Source: PLoS One. 2015 Feb 2;10(2):e0115557. doi: 10.1371/journal.pone.0115557 (PMC4313934; doi:10.1371/journal.pone.0115557)

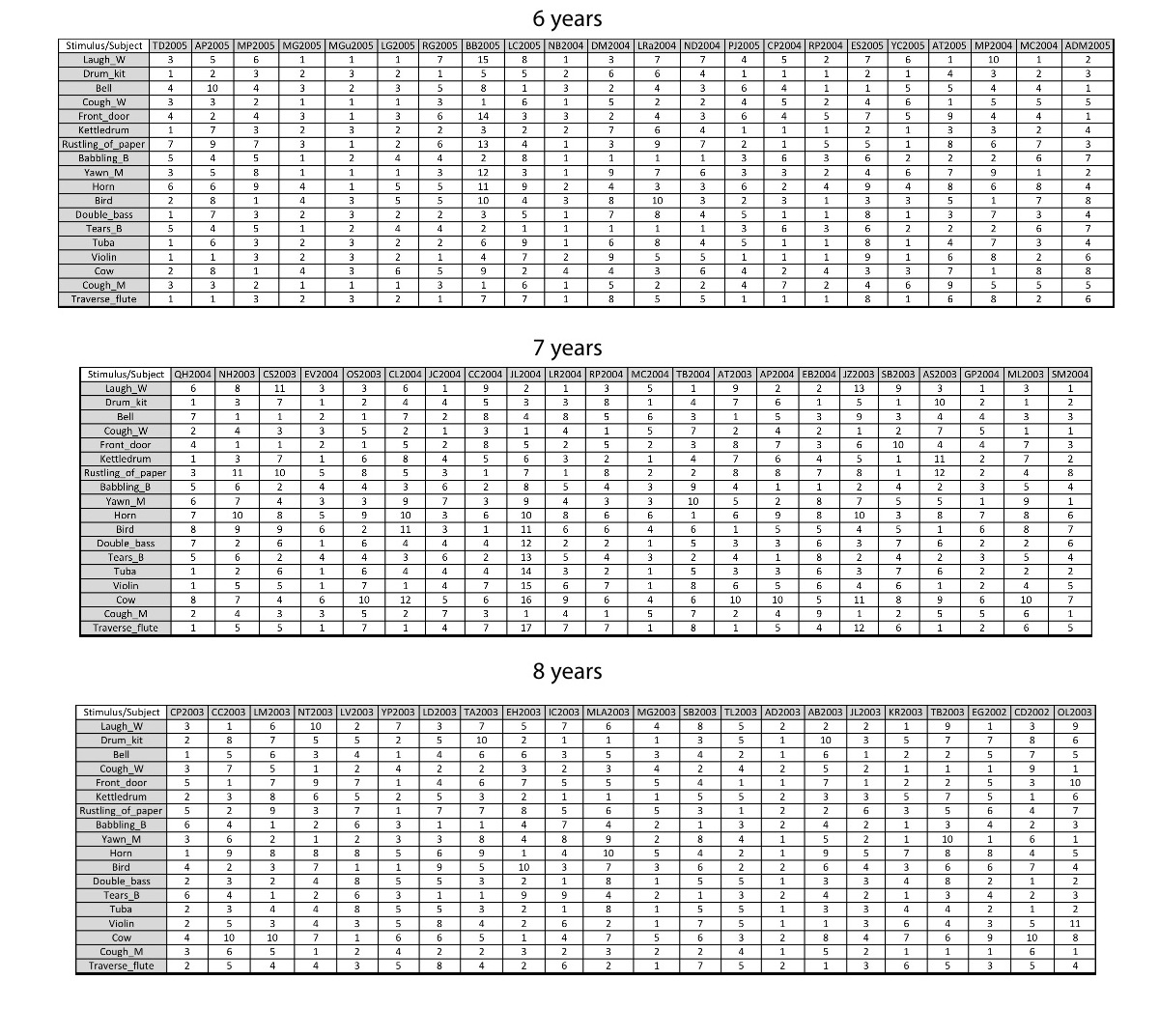

Supplement: S1 Table — Results for 6, 7 and 8 years old participants. Each column represents the classification performed by an individual subject, and in each line, we are providing the category number in which the individual sounds have been pooled together. In consequence, for each individual, the highest number corresponds to the maximum number of classes performed by the subject. (TIFF) [file pone.0115557.s001.tiff]

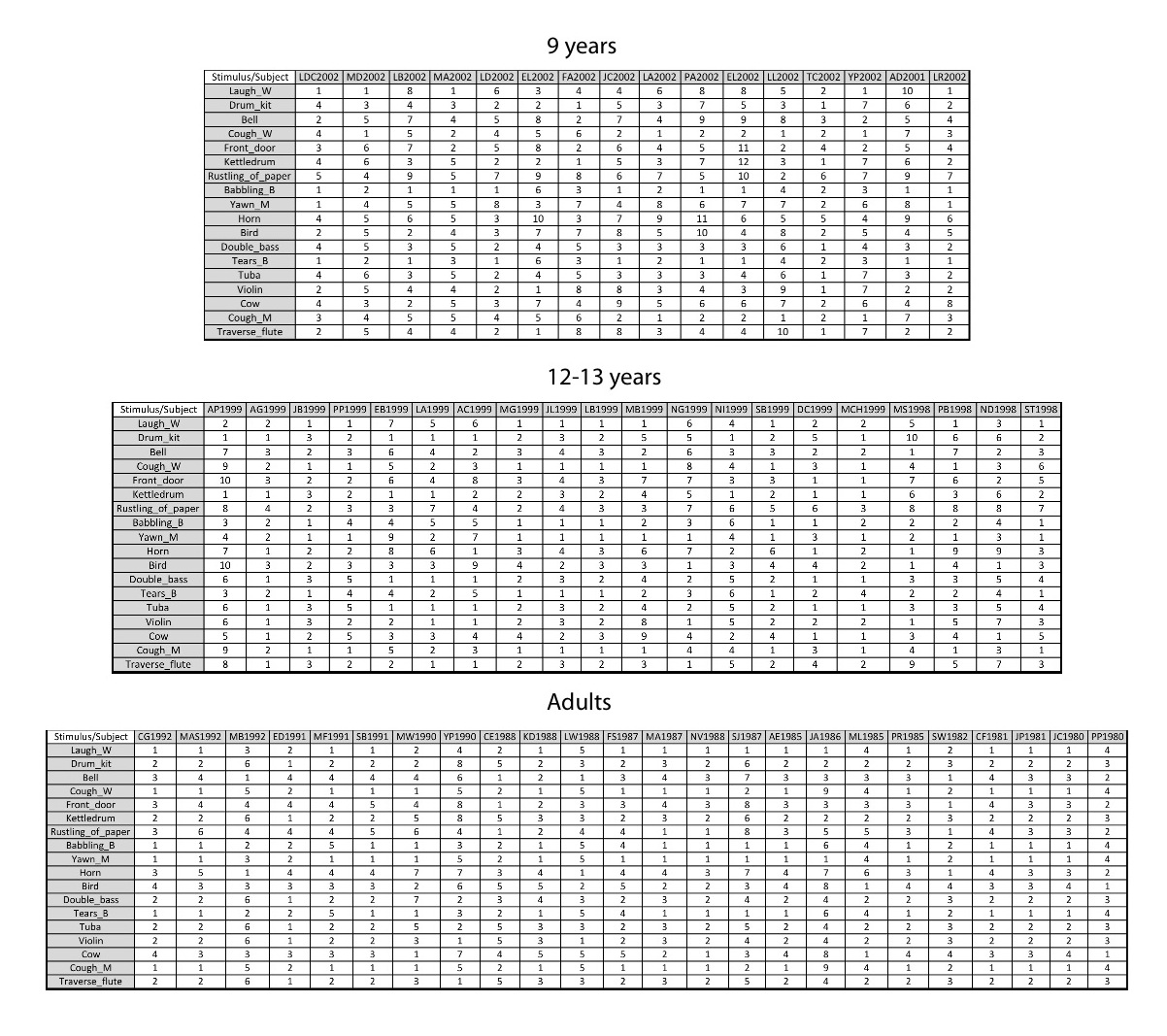

Supplement: S2 Table — Results for 9, 12–13 years old participants and adults. Same convention as in S1 Table. (TIFF) [file pone.0115557.s002.tiff]
